# Alternating binding and p97-mediated dissociation of SDS22 and I3 recycles active PP1 between holophosphatases

**DOI:** 10.1073/pnas.2408787121

**Published:** 2024-08-29

**Authors:** Anja F. Kueck, Johannes van den Boom, Sandra Koska, David Ron, Hemmo Meyer

**Affiliations:** ^a^Molecular Biology I, Center of Medical Biotechnology, Faculty of Biology, University of Duisburg-Essen, 45141 Essen, Germany; ^b^Cellular Pathophysiology and Clinical Biochemistry, Cambridge Institute for Medical Research, University of Cambridge, Cambridge CB2 0XY, United Kingdom

**Keywords:** protein phosphatase-1, AAA+ ATPase, VCP, protein unfolding, Förster resonance energy transfer

## Abstract

Protein phosphatase-1 catalytic subunit (PP1) joins diverse targeting subunits to form holophosphatases that regulate many cellular processes. Newly synthesized PP1 is known to be transiently sequestered in an inhibitory complex with Suppressor-of-Dis2-number-2 (SDS22) and Inhibitor-3 (I3), which is disassembled by the ATPases Associated with diverse cellular Activities plus (AAA+) protein p97. Here, we show that the SDS22-PP1-I3 complex also acts as a thermodynamic sink for mature PP1 and that cycles of SDS22-PP1-I3 formation and p97-driven disassembly regulate PP1 function and subunit exchange beyond PP1 biogenesis. Förster Resonance energy transfer (FRET) analysis of labeled proteins in vitro revealed that in the p97-mediated disassembly step, both SDS22 and I3 dissociate concomitantly, releasing PP1. In presence of a targeting subunit, for instance Growth Arrest and DNA Damage-inducible protein 34 (GADD34), liberated PP1 formed an active holophosphatase that dephosphorylated its substrate, eukaryotic translation initiation factor 2 alpha (eIF2α). Inhibition of p97 results in displacement of the GADD34 targeting subunit by rebinding of PP1 to SDS22 and I3 indicating that the SDS22-PP1-I3 complex is thermodynamically favored. Likewise, p97 inhibition in cells causes rapid sequestration of PP1 by free SDS22 and I3 at the expense of other subunits. This suggests that PP1 exists in a steady state maintained by spontaneous SDS22-PP1-I3 formation and adenosine triphosphate (ATP) hydrolysis, p97-driven disassembly that recycles active PP1 between different holophosphatase complexes to warrant a dynamic holophosphatase landscape.

## Significance

A limiting pool of protein phosphatase-1 catalytic subunits (PP1) needs to dynamically form holophosphatases with alternative regulatory subunits to dephosphorylate diverse cellular targets in varying physiological circumstances. During biogenesis, PP1 is trapped in a complex with inhibitory partners Suppressor-of-Dis2-number-2 (SDS22) and Inhibitor-3 (I3), which is disassembled by the ATPases Associated with diverse cellular Activities plus (AAA+) protein p97 to allow formation of PP1 holoenzymes with targeting subunits. Here, we report that this mechanism extends to mature PP1, whose availability to join alternative targeting subunits is actively maintained by cycles of mass action-driven formation of SDS22-PP1-I3 complexes and their p97- driven disassembly to release PP1 for holophosphatase formation. This suggests an energy-driven process that facilitates dynamic exchange of PP1 to match holophosphatase composition to changing physiological conditions.

Protein phosphatase-1 is expressed in all eukaryotic cells and catalyzes a large fraction of all phospho-serine/threonine dephosphorylation reactions (1). A common catalytic subunit [protein phosphatase-1 catalytic subunit (PP1) from hereon] participates in functionally diverse reactions by forming holophosphatases with diverse regulatory subunits that serve as targeting factors and substrate specifiers (2).

Two conserved proteins, SDS22 (also called PPP1R7, or Sds22 in yeast) and Inhibitor-3 (I3, also called PPP1R11, or Ypi1 in yeast), stand out among the PP1 partners as they trap newly synthesized PP1 in an inactive ternary complex (3–6). I3 is bound through a short linear arginine-valine-any amino acid-phenylalanine (RVXF) motif to a site on the surface of PP1, which is shared by many regulatory subunits (4). PP1 has two metal ions bound in the catalytic site, M1 and M2 (7, 8). I3 covers the catalytic region and makes contact with one of the two metal ions, M2, in the catalytic site of PP1 (9, 10). In contrast, SDS22 binds via a large surface of leucine-rich repeats to a region outside the catalytic region, but this binding affects the interaction site for the other metal ion, M1 (11–13).

During PP1 biogenesis, the SDS22-PP1-I3 complex forms transiently and is disassembled in an energy-consuming process by the AAA+ ATPase p97 (also called VCP for Valosin-containing protein or, in yeast, Cdc48). Disassembly is believed to allow PP1 association with other subunits and thus formation of active PP1 holophosphatases (5). p97 is a homohexamer in which the AAA domains form two stacked rings with a central channel while the regulatory N-domains are positioned at the periphery of the hexamer. In contrast to p97’s function in the ubiquitin–proteasome system, in which substrate proteins are recruited to p97 by the Ufd1-Npl4 ubiquitin adapter (14–16), SDS22-PP1-I3 disassembly is ubiquitin-independent and supported by the p37 substrate adapter (also called UBXN2B) or the related UBXN2A (5, 17). p37-assisted loading of the SDS22-PP1-I3 complex onto p97 locks SDS22 into a groove of the p97 N-domain and partially inserts I3 into the central channel of p97 (18, 19). ATP-driven threading of I3 through the channel then strips I3 off PP1 and leads to disassembly of the whole complex (5).

However, it has not been shown directly that SDS22-PP1-I3 disassembly, as proposed, can actually lead to formation of an active PP1 holoenzyme. Moreover, it is unclear whether SDS22-PP1-I3 formation and disassembly is restricted to newly synthesized PP1 during PP1 biogenesis, or whether it also regulates PP1 and PP1 holophosphatase formation later in the life-cycle of PP1. Here, we reconstituted p97-driven PP1 subunit exchange and formation of an active PP1 holoenzyme from purified components demonstrating directly that p97 drives formation of active PP1 holophosphatases. Moreover, we provide evidence that regulation by SDS22 and I3 extends beyond PP1 biogenesis and that cellular PP1 exists in a steady state between SDS22-PP1-I3 complex formation and its p97-driven disassembly, which recycles PP1 between holophosphatases.

## Results

### Dissociation of SDS22 and I3 from PP1 Is Coupled.

**Fig. 1.**
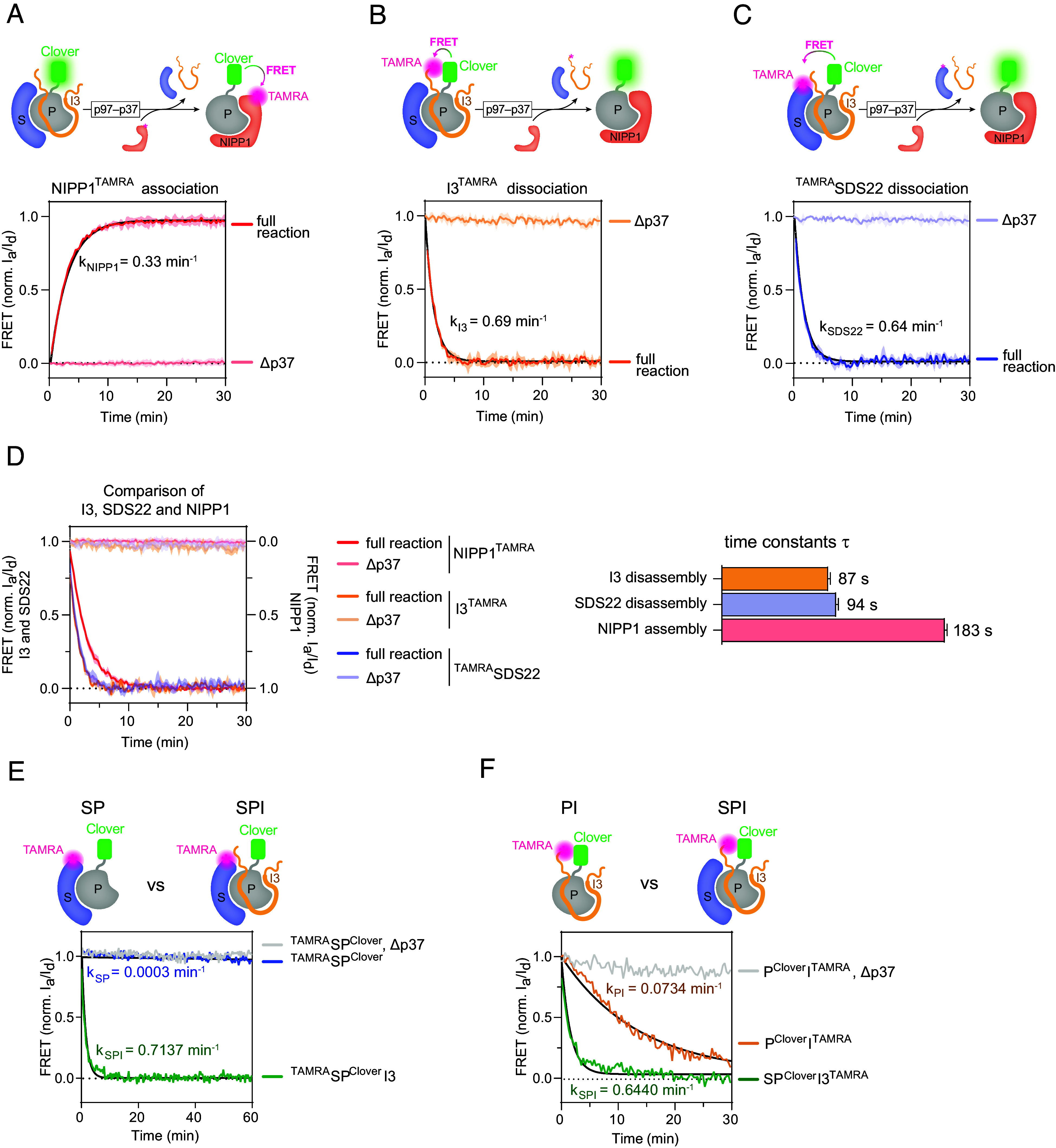
Detailed FRET analysis of p97-mediated PP1 subunit exchange reaction. (*A*–*C*) Variations of the PP1 subunit exchange reaction. PP1 fused to FRET donor Clover was generated in complex with SDS22 and I3. The FRET acceptor TAMRA was conjugated to either I3 or SDS22, or to the alternative subunit NIPP1, as indicated. The SDS22-PP1-I3 complex (160 nM) was incubated with p97 (160 nM) and the p37 adapter protein (480 nM) in the presence of a slight excess of the alternative subunit NIPP1 (240 nM) over PP1. Reactions lacking the adapter (Δp37) served as negative controls. Shown are the means of the normalized ratios of acceptor and donor fluorescence ± SD, n = 3. Rate constants from exponential fit were k_NIPP1_ = 0.33 ± 0.0026 min^–1^, k_I3_ = 0.69 ± 0.0012 min^–1^, and k_SDS22_ = 0.64 ± 0.0105 min^–1^. (*D*) Overlay of the reaction curves determined in *A*–*C*. The NIPP1 curve was inversed for clarity. Note concomitant dissociation of SDS22 and I3 followed by association of NIPP1. Curves were fitted with exponential curves, yielding time constants (τ) for I3 disassembly (1.46 ± 0.025 min^−1^), SDS22 disassembly (1.57 ± 0.03 min^−1^), and NIPP1 assembly (3.05 ± 0.024 min^−1^). Curves, n = 3 ± SD; time constants and fit values, n = 3 ± SEM. (*E* and *F*) Efficient SDS22 and I3 dissociation from PP1 by p97 requires the full SDS22-PP1-I3 complex (SPI). Subunit exchange reactions as in *A*−*C* of the ternary SDS22^TAMRA^-PP1-I3 and SDS33-PP1-I3^TAMRA^ were compared with binary complexes lacking the respective other unlabeled subunit (SP and PI). Note that SDS22^TAMRA^-PP1 was not dissociated, and that PP1-I3^TAMRA^ was disassembled only at low rate. Shown are the representative normalized ratios of acceptor and donor fluorescence of three independent experiments. Rate constants (±SEM) from exponential fit were k_SP_ = 0.0003 ± 0.0001 min^−1^, k_SPI_ (TAMRA on SDS22) = 0.7137 ± 0.0204 min^−1^, k_PI_ = 0.0734 ± 0.0003 min^−1^, and k_SPI_ (TAMRA on I3) = 0.6440 ± 0.0267 min^−1^.

To explore PP1 activation and its relation to SDS22 and I3, we first aimed to assess the SDS22-PP1-I3 complex and its disassembly in more detail. Disassembly of the SDS22-PP1-I3 can be observed indirectly by monitoring association of the alternative subunit NIPP1 to PP1 that is released during the reaction using FRET (18). In this setup, SDS22-PP1-I3 is used with the FRET donor Clover fused to PP1 and incubated with an excess of NIPP1 that is labeled with the FRET acceptor TAMRA. In the presence of p97 along with its adapter p37, the FRET signal rapidly increases (Fig. 1*A*) demonstrating binding of NIPP1 to PP1, and indicating p97-mediated dissociation of I3 and SDS22 from PP1. In contrast, when the adapter p37 is omitted so that p97 cannot target SDS22-PP1-I3, no binding of NIPP1 to PP1 occurs, as expected, because PP1 remains sequestered by SDS22 and I3 (Fig. 1*A*). In this and following in vitro assays, the PP1γ isoform (also called PPP1CC) was used.

To monitor dissociation of SDS22 and I3 more directly and to explore how they are linked, we generated complexes of PP1^Clover^ with SDS22 and I3, in which either SDS22 or I3 was labeled with TAMRA (*SI Appendix*, Fig. S1) resulting in FRET between the two chromophores as long as the complex stayed formed. Incubation of SDS22^TAMRA^-PP1^Clover^-I3 or SDS22-PP1^Clover^-I3^TAMRA^ with p97 and p37 in the presence of unlabeled NIPP1 led to rapid loss of FRET (Fig. 1 *B* and *C* and *SI Appendix*, Fig. S2 *A* and *B*) demonstrating that either subunit was released from PP1 and, thus, that disassembly of the SDS22-PP1-I3 complex occurred. Relative changes in donor (Clover) and acceptor (TAMRA) fluorescence were comparable and donor fluorescence did not change during the reaction in the absence of the TAMRA label, as expected (*SI Appendix*, Fig. S2 *C* and *D*). SDS22-PP1-I3 complex disassembly and NIPP1 binding was confirmed by coimmunoprecipitation (*SI Appendix*, Fig. S2*E*). Complex disassembly was strictly dependent on p97 activity, because disassembly did not occur when the p37 adapter was omitted (Fig. 1 *B* and *C*). Even at long-term incubations over a period of 18 h and at sixfold excess of NIPP1, SDS22-PP1-I3 stayed largely stable in the absence of p97-p37, and NIPP1 did not compete with the SDS22 and I3 subunits (*SI Appendix*, Fig. S2 *F* and *G*). Comparison between the rates of SDS22 and I3 dissociation from PP1 revealed that both partners dissociated simultaneously (Fig. 1*D*) and this was true over a range of disassembly rates at different p97 concentrations (*SI Appendix*, Fig. S2*H*). We previously established that the NIPP1 association rate reaches a maximum at the concentration used here (18). NIPP1 associated with PP1 with a slight delay, as illustrated by the different time constants τ of the reactions, showing that SDS22 and I3 dissociation is not driven by NIPP1 binding (Fig. 1*D*).

I3 is the direct substrate of p97 whose threading through the p97 channel strips I3 from PP1 (18, 19). SDS22 subunit contributes to targeting of SDS22-PP1-I3 to p97 by direct interaction of SDS22 with the p97 N-domain (18, 19). How I3 stripping by p97 induces SDS22 dissociation is not understood but may involve a direct interaction between I3 and SDS22 (20). To elucidate the reciprocal relationship further, we generated the binary SDS22^TAMRA^-PP1^Clover^ and PP1^Clover^-I3^TAMRA^ complexes in the absence of the respective other partner and compared disassembly with that of the complete SDS22^TAMRA^-PP1^Clover^-I3 or SDS22-PP1^Clover^-I3^TAMRA^ complexes. SDS22^TAMRA^-PP1^Clover^ was not disassembled in the absence of I3 showing that I3 must be targeted by p97 to also dissociate SDS22 (Fig. 1*E*). PP1^Clover^-I3^TAMRA^ was disassembled in the absence of SDS22, but at a rate almost an order of magnitude lower as compared to the full SDS22-PP1^Clover^-I3^TAMRA^ complex (Fig. 1*F*) consistent with SDS22 helping to target the complex to p97. Thus, the relevant substrate of p97 is the PP1 complex with both SDS22 and I3, and both subunits are dissociated essentially simultaneously, likely in a highly cooperative process.

### The SDS22-PP1-I3 Complex Is Thermodynamically Favored over Holoenzymes.

**Fig. 2.**
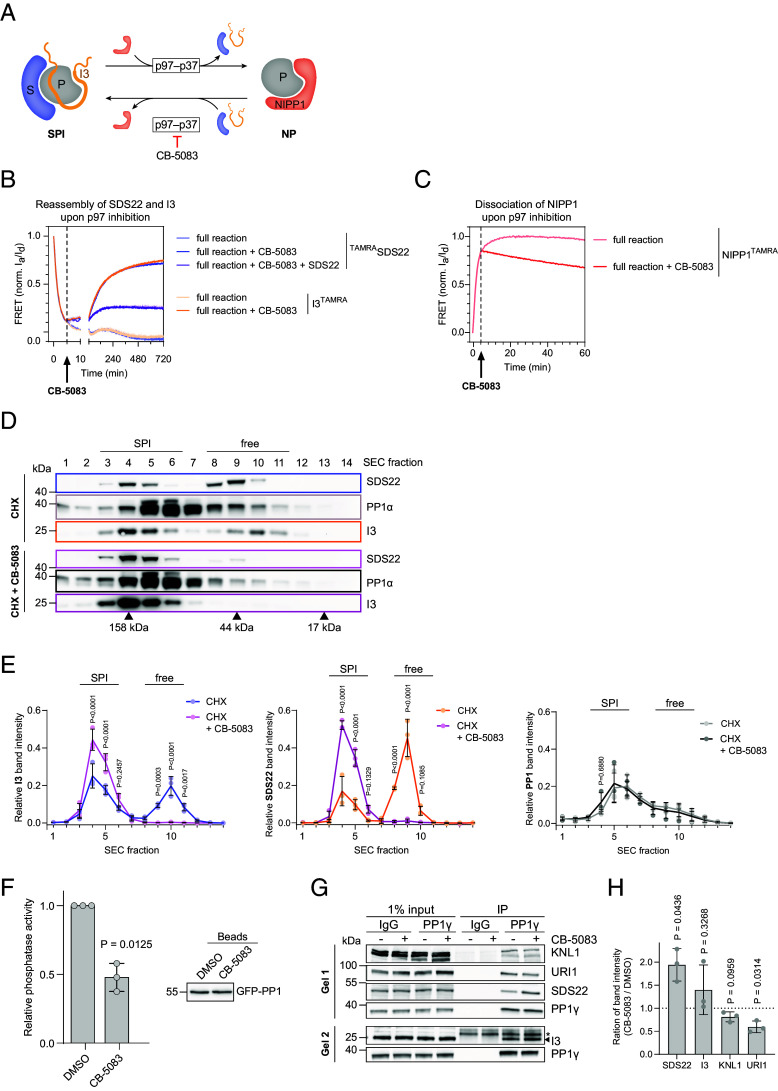
Reformation of SDS22-PP1-I3 is thermodynamically favored in the absence of p97 activity. (*A*) Model for p97-mediated subunit exchange and its reversal upon treatment with p97 inhibitor CB-5083. (*B*) Subunit exchange reactions as followed by FRET with TAMRA-labeled SDS22 or I3 subunits in the presence of unlabeled NIPP1. CB-5083 or vehicle alone was added after 5 min once SDS22-PP1-I3 disassembly was nearly completed. Note that labeled SDS22 and I3 rebind when p97 is inhibited, and that rebinding of SDS22 was reduced by addition of competing unlabeled SDS22 (10-fold excess over ^TAMRA^SDS22). Shown are the normalized ratios of acceptor and donor fluorescence of two independent experiments (three for SDS22 with CB-5083) ± SD. (*C*) Reactions as in *B* but following TAMRA-labeled NIPP1 association. Note that NIPP1 dissociates upon p97 inhibition. Representative experiment of three repeats with similar outcome. (*D*) Size exclusion chromatography of lysates of cells treated with CB-5083 or vehicle alone for 70 min while synthesis of PP1 was blocked by cycloheximide (CHX). Western blot with indicated antibodies. Note shift of monomeric SDS22 and I3 (free) to the PP1-bound pool (SPI) upon p97 inhibition. Indicated molecular weight references were obtained using protein standard. Representative blots of three repeats with similar outcome. (*E*) Quantification of *D*. Band intensities were quantified and presented as a fraction of each protein. N = 3 ± SD. *P* values were determined by two-way ANOVA with Šídák’s multiple comparisons test. *P* values > 0.7 are omitted. SPI, SDS22-PP1-I3 complex; free, free SDS22, PP1, or I3 proteins. (*F*) CB-5083 treatment causes a drop in cellular PP1 activity. Green fluorescent protein (GFP)-PP1 was isolated from stable HeLa cells treated with CB-5083 or vehicle alone for 2 h in the presence of CHX. Phosphatase activity determined by OMFP dephosphorylation assay and normalized to immunoprecipitated GFP-PP1 as quantified by western blot for each experiment. Mean ± SD from three biological replicates. The *P* value was determined by the one-sample *t* test. (*G*) Reduced binding of holophosphatase subunits to PP1 upon p97 inhibition. HeLa cells were treated as indicated for 70 min. Endogenous PP1 was immuno-isolated from lysates and associated subunits detected with indicated antibodies. Representative blots of three repeats with similar outcome. (*H*) Quantification of western blot signal intensities of indicated proteins in *G*. N = 3 ± SD. *P* values were determined by the one-sample *t* test against a theoretical mean of 1.0. Normalization to values without CB-5083.

The experiments above illustrate a PP1 subunit exchange reaction in which SDS22 and I3 are displaced from PP1 by p97 to allow binding of NIPP1 to PP1 (Fig. 2*A*). We noted that net disassembly of the SDS22-PP1-I3 complex, as detected by FRET, increased with increasing concentrations of NIPP1 (*SI Appendix*, Fig. S3 *A* and *B*). This indicates that association of PP1 with NIPP1 lowers its rate of rebinding to SDS22 and I3 after p97-mediated dissociation of the SDS22-PP1-I3 complex. These observations suggest a kinetically imposed ATP hydrolysis-dependent steady state affecting the partitioning of PP1 between the two complexes. By enhancing the pool of free PP1, p97 favors the formation of a PP1-NIPP1 complex. We therefore asked how this steady state is affected by p97 inhibition.

Of note, upon addition of the p97 inhibitor CB-5083 to reactions in which the subunit exchange from SDS22 and I3 to NIPP1 was largely completed, the interaction pattern was reversed with reestablishment of SDS22-PP1-I3 at the expense of NIPP1-PP1 complexes (Fig. 2*B*). This was confirmed in reactions in which the TAMRA label was placed on either SDS22 or I3 (Fig. 2*B*). The observed increase in FRET after p97 inhibition was reduced by addition of competing unlabeled SDS22 indicating that the signal change indeed stemmed from rebinding of labeled SDS22 (Fig. 2*B*). Conversely and consistent with that notion, TAMRA-labeled NIPP1, which associated with PP1 while p97 was active, was gradually displaced once p97 was inhibited (Fig. 2*C*). p97-mediated SDS22-PP1-I3 disassembly and subsequent rebinding of SDS22 and I3 to PP1 upon p97 inhibition was confirmed by coimmunoprecipitation of PP1 (*SI Appendix*, Fig. S3*C*). Notably, this relationship did not represent simple competition between NIPP1 and SDS22 or I3, because exchange of SDS22 and I3 with NIPP1 on PP1 strictly required p97 activity, whereas displacement of NIPP1 by SDS22 and I3 occurred in the absence of p97 activity (Fig. 2 *B* and *C*). Thus, in the presence of SDS22 and I3, the SDS22-PP1-I3 complex is thermodynamically favored, whereas binding of an alternative subunit to PP1 requires energy-driven displacement of SDS22 and I3 by p97.

In cells, SDS22 and I3 exist in two pools, the monomeric forms and the pool bound to PP1 (5). We previously found that inhibition of p97 in cells leads to a shift of both subunits to the PP1-bound form, which we attributed to sequestration of SDS22 and I3 by newly synthesized PP1 (5). However, the observation that SDS22 and I3 form a complex with PP1 released from NIPP1, suggests that in cells, too, mature folded PP1 may also form a complex with SDS22 and I3. Indeed, we note that the partitioning of SDS22 and I3 away from the free pool to a complex with PP1 upon p97 inhibition also occurred when synthesis of new PP1 was blocked by cycloheximide (CHX), and was observed during treatments as short as 70 min (Fig. 2 *D* and *E* and *SI Appendix*, Fig. S3 *D* and *E*). Consistent with more PP1 being sequestered by the inhibitory subunits, PP1 immuno-isolated from p97-inhibited cells, was less active compared to isolates from control cells even when synthesis of new PP1 was inhibited (Fig. 2*F*). Thus, SDS22 and I3 not only bind to newly synthesized PP1 during PP1 biosynthesis but also to mature PP1 during later stages of the PP1 life cycle. Moreover, the ratio of SDS22 and I3-bound PP1 and free PP1 is in a steady state which is actively maintained by cycles of p97-mediated disassembly of SDS22-PP1-I3 likely to make PP1 available for the formation of active PP1 holophosphatases. In line with that notion, we observed reduced cellular binding of PP1 to exemplary holophosphatase subunits such as URI1 and KNL1, as shown by PP1 coimmunoprecipitation from cells (Fig. 2 *G* and *H*). We confirmed the ability of SDS22 and I3 to displace holophosphatase subunits such as NIPP1 from PP1 in an in vitro experiment in which preformed NIPP1^TAMRA^-PP1^Clover^ complex was incubated with SDS22-PP1-I3 and p97-p37. In accordance with our model, SDS22 and I3, which were released from PP1 by p97, displaced NIPP1 as detected by the loss of FRET (*SI Appendix*, Fig. S3 *F*–*H*).

### Disassembly of SDS22-PP1-I3 by p97 Releases Active PP1.

**Fig. 3.**
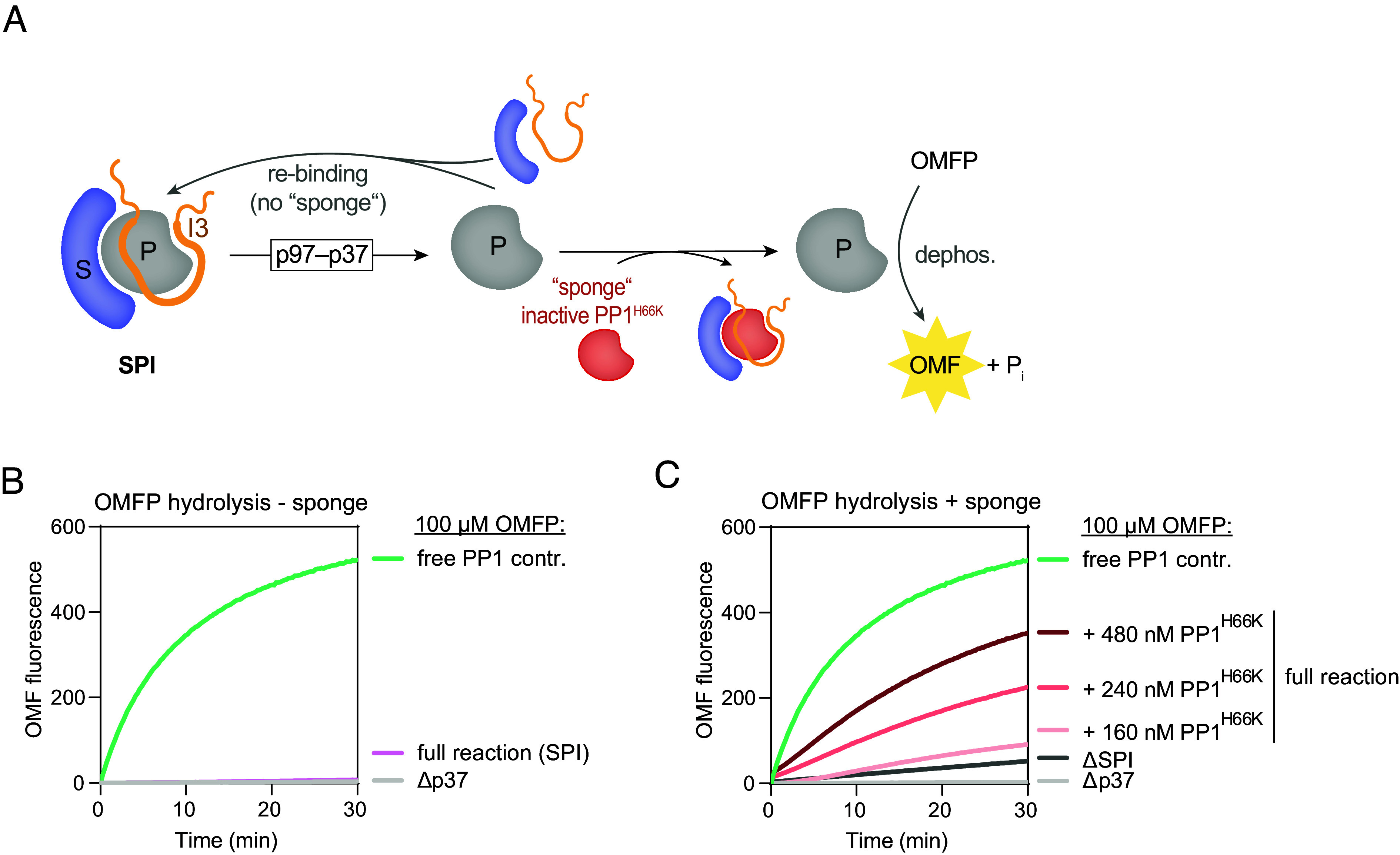
Disassembly of SDS22-PP1-I3 releases active PP1. (*A*) Model for p97-mediated activation of PP1. p97-p37 disassemble SDS22-PP1-I3, but SDS22 and I3 rebind PP1 after disassembly thus keeping PP1 inactive. The inactive PP1^H66K^ added to the reaction acts as sponge to bind SDS22 and I3 and allows free PP1 to dephosphorylate fluorogenic OMFP. (*B*) Fluorescence spectrometry of OMF. OMFP is readily converted to fluorescent OMF by free PP1. Note that incubation of SDS22-PP1-I3 (SPI) with proficient p97 and p37 (full reaction) in the absence of PP1^H66K^ does not lead to net production of active PP1. The Δp37 control denotes a full reaction lacking the p37 adapter required for p97 activity. Shown are the representative raw OMFP fluorescence intensities of three independent experiments with similar outcome. (*C*) As in *B* but with increasing concentrations of the PP1^H66K^ sponge, or PP1^H66K^ alone, as indicated. Free PP1 curve from *B* was included as reference. All protein components were preincubated before addition of OMFP, and the reaction started by adding ATP. Shown are the representative raw OMFP fluorescence intensities of three independent experiments with similar outcome.

The model for SDS22-PP1-I3 formation and disassembly outlined above entails that disassembly of the SDS22-PP1-I3 complex by p97 leads to release of active PP1. To confirm this directly, we coupled the p97-mediated SDS22-PP1-I3 disassembly reaction to dephosphorylation of the small molecule substrate 3-O-methylfluorescein phosphate bis-cyclohexylammonium salt (OMFP), which generates fluorescent OMF (Fig. 3*A*). Free PP1 dephosphorylated OMFP leading to an increase in OMF fluorescence (Fig. 3*B*). In contrast, PP1 supplied in the SDS22-PP1-I3 complex was inactive as expected. Intriguingly, addition of p97 and p37 did not result in dephosphorylation of OMFP even though conditions were proficient for SDS22-PP1-I3 disassembly (Fig. 3*B*). This could be explained if the kinetics of p97-mediated dissociation and complex reformation were skewed in favor of the latter, limiting the pool of free PP1. The lack of any detectable activity under these dynamic conditions may additionally be due to the time needed for repositioning the metal ion in the catalytic center that is absent when SDS22 is bound (13). Thus, we sought to limit rebinding of SDS22 and I3 to the released PP1 by adding an excess of inactive PP1^H66K^ variant to act as a “sponge” that sequesters SDS22 and I3 (Fig. 3*A*). Addition of PP1^H66K^ to the disassembly reaction led to dephosphorylation of OMFP in a PP1^H66K^ concentration-dependent manner in conditions proficient for p97-mediated SDS22-PP1-I3 disassembly, but not when the p37 adapter was omitted or when PP1^H66K^ was added alone (Fig. 3*C*). Thus, dissociation of SDS22 and I3 from PP1 by p97 promotes release of active PP1.

### Coupling of SDS22-PP1-I3 Disassembly with Formation of the Active PP1-GADD34 Holoenzyme.

**Fig. 4.**
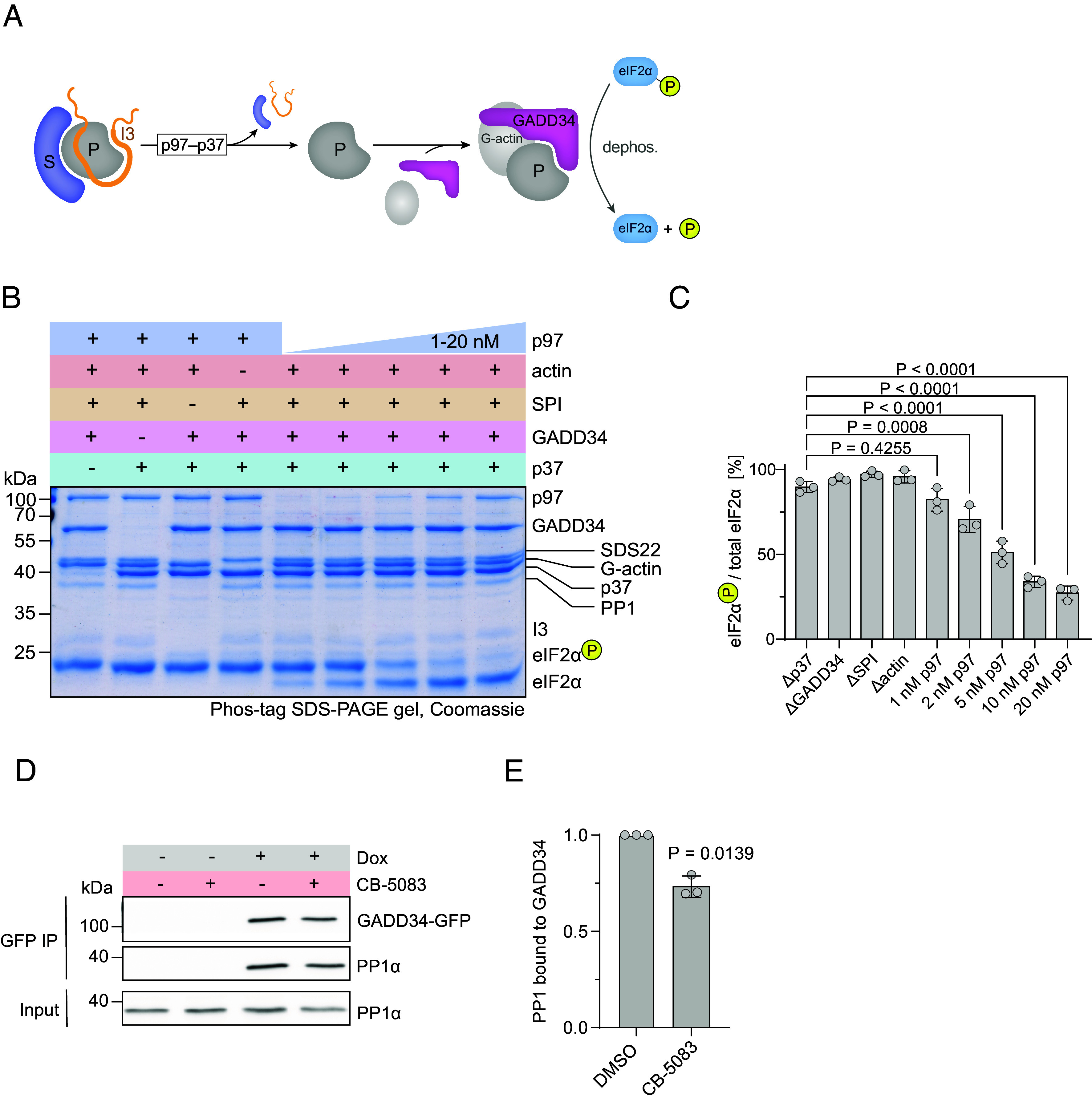
SDS22-PP1-I3 disassembly coupled to formation of an active PP1-GADD34 holoenzyme. (*A*) Model for p97-mediated disassembly of SDS22-PP1-I3 coupled to formation of active PP1-GADD34 that dephosphorylates eIF2α. G-actin is required for PP1-GADD34 activity. (*B*) Coupled PP1 subunit exchange and eIF2α dephosphorylation reactions with purified proteins as analyzed in PhosTag gels. eIF2α was phosphorylated beforehand by incubation with PERK and reisolated. Indicated protein combinations were incubated for 10 min. Note that eIF2α dephosphorylation required p97-p37 along with GADD34 and G-actin when SDS22-PP1-I3 (SPI) was provided as a source of PP1, and increased with increasing p97 concentrations. Representative gel of three repeats. (*C*) Quantification of *B*. eIF2α-P present at the end of the reaction for three experiments was normalized to one ΔGADD34 sample, with eIF2α-P + eIF2α = 100%. Shown are means and SD of the absolute values (n = 3). *P* values were determined by one-way ANOVA with Šídák’s multiple comparisons test. *P* values > 0.7 are omitted. (*D*) p97 activity is required for efficient PP1-GADD34 formation in cells after GADD34 induction. GADD34-GFP expression was induced (DOX) for 5 h in a stable HeLa cell line and cells treated with CB-5083 or vehicle alone for 3 h. GADD34-GFP was immunoprecipitated from lysates and associated PP1 analyzed by western blotting with indicated antibodies. Representative gel of three biological replicates. (*E*) Quantification of band intensities in *D*. Mean ± SD from three biological replicates. The *P* value was determined by the one sample *t* test.

To efficiently dephosphorylate phosphoproteins, PP1 forms holoenzymes with substrate-specifying subunits. We therefore next aimed to recapitulate PP1 holoenzyme formation and activation with a relevant substrate specifier (Fig. 4*A*). We chose GADD34 (also called PPP1R15A) a regulator of the integrated stress response (ISR). Stress-activated kinases such as protein kinase R-like endoplasmic reticulum kinase (PERK) phosphorylate the translation initiation factor, eIF2α at serine-51 to attenuate protein synthesis (Fig. 4*A*). GADD34 is encoded by an ISR target gene. By binding to PP1 and dephosphorylating eIF2α, GADD34 closes a negative feedback loop in the ISR. We generated an active fragment of GADD34, comprising relevant residues 513 to 636, fused to maltose binding protein (MBP) (21) (*SI Appendix*, Fig. S4*A*). Direct TAMRA-labeling of GADD34 failed because the conjugation induced GADD34 aggregation. However, we confirmed p97-mediated binding of GADD34 to PP1 in the p97-mediated subunit exchange reaction indirectly, because GADD34 increased net disassembly of the SDS22-PP1-I3 complex by sequestering PP1, and this was dependent on the GADD34 concentration (*SI Appendix*, Fig. S4 *B* and *C*), demonstrating that GADD34 replaced SDS22 and I3 on PP1. Moreover, once p97 was inhibited in this reaction, the SDS22 and I3 subunits bound back to PP1 (*SI Appendix*, Fig. S4*D*) indicating that, in the absence of p97 activity, GADD34, like NIPP1, is displaced by mass action with SDS22 and I3.

We next asked whether subunit exchange resulting in formation of the GADD34-PP1 complex led to specific activation with respect to eIF2α dephosphorylation. Purified eIF2α was phosphorylated by PERK and reisolated (*SI Appendix*, Fig. S5*A*). Phospho-eIF2α was then incubated in various protein combinations and the phosphorylation status assayed in Phos-tag gels. G-actin, a targeting cofactor of eIF2α to GADD34, was included in these reactions (22, 23). PP1 supplied in complex with SDS22 and I3 did not dephosphorylate eIF2α, as expected, even in the presence of p97-p37 (Fig. 4 *B* and *C*), indicating that GADD34 was required for eIF2α dephosphorylation. Conversely, addition of GADD34 alone to SDS22-PP1-I3 in the absence of p97 activity did not result in eIF2α dephosphorylation (Fig. 4 *B* and *C*) confirming that GADD34 cannot displace SDS22 and I3 simply by mass action. Introduction of p97-p37 promoted robust dephosphorylation of eIF2α (Fig. 4 *B* and *C*), indicating that an active PP1-GADD34 complex formed. As expected, the rate of dephosphorylation was dependent on the concentrations of both p97 and SDS22-PP1-I3 as the source of PP1, and required G-actin (Fig. 4 *B* and *C* and *SI Appendix*, Fig. S5*B*). Thus, in vitro, p97 releases PP1 from the inhibitory partners SDS22 and I3 and thereby mediates formation of an active PP1-GADD34-G-actin holoenzyme that is proficient in substrate dephosphorylation.

Next, we aimed to examine p97’s role in PP1-GADD34 holoenzyme formation in cells. While GADD34-mediated eIF2α dephosphorylation powerfully antagonizes the activity of ISR markers (24), the central role of p97 in protein quality control, which stands upstream of ISR activating kinases (e.g., PERK), confounds interpretation of the impact of p97 inhibition on ISR markers. We therefore resorted to a doxycycline-inducible system for GADD34 expression in a stable cell line. PP1 readily bound newly expressed GADD34-GFP after induction as detected by coimmunoprecipitation of GADD34-GFP (Fig. 4 *D* and *E*). Of note, inhibition of p97 partially reduced PP1-GADD34 formation (Fig. 4 *D* and *E*) consistent with a role for p97 in the release of PP1 from the SDS22-PP1-I3 complex to allow efficient GADD34 binding in cells.

## Discussion

**Fig. 5.**
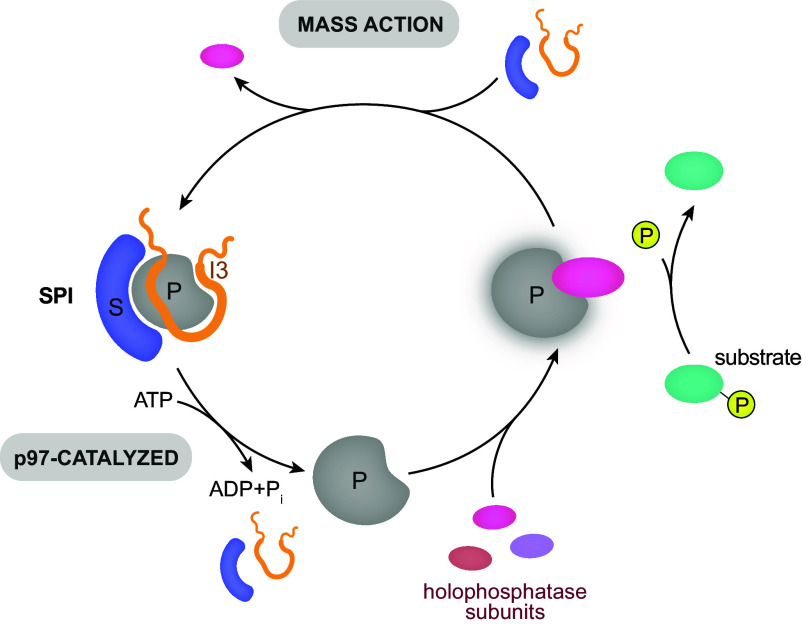
Model for regulation of PP1 by cycling between SDS22 and I3 binding and release. The SDS22-PP1-I3 complex serves as a thermodynamic sink for free PP1, with SDS22 and I3 even displacing holoenzyme subunits. Once formed, p97 consumes ATP to disassemble the SDS22-PP1-I3 complex. PP1 is thus made available for formation of active holoenzymes and substrate dephosphorylation. When p97 is compromised, PP1 is sequestered in the inactive SDS22-PP1-I3 complex. This cycle protects cells from free PP1 and ensures a dynamic formation of holophosphatase complexes.

Previous work suggested that SDS22 and I3 function as maturation factors consistent with their transient binding during PP1 biogenesis and the fact that they are inhibitory, yet essential for PP1 activity (3–5). In this study, we provide evidence of a role for SDS22 and I3 in intracellular phosphoprotein dephosphorylation that goes beyond PP1 biogenesis. We show that even after biogenesis, PP1 binds SDS22 and I3 and undergoes cycles of SDS22-PP1-I3 formation and dissociation resulting in a steady state between a free pool of PP1 available for holoenzyme formation and a pool of PP1 locked in the inhibitory complex (see model in Fig. 5). This steady state is maintained by formation of SDS22-PP1-I3 that is driven by mass action and can compete effectively with other subunits for a limited pool of PP1. Conversely, once bound, SDS22 and I3 are not displaced from PP1 even in presence of large excess of alternative subunits and incubation for many hours due to the high stability of SDS22-PP1-I3. Instead, SDS22-PP1-I3 dissociation is an energy-dependent process and requires the strong unfolding enzyme p97 (5, 18). This suggests that the SDS22-PP1-I3 complex acts as a thermodynamic sink, possibly to protect cells from uncontrolled activity of free PP1 from dissociating holophosphatases. The SDS22-PP1-I3 pool then acts as a PP1 storage for new holophosphatases consistent with our finding that p97 is needed for efficient holophosphatase formation in cells. In addition, we noted that SDS22 and I3 even displace subunits in PP1 holophosphatases by sequestering PP1. This indicates that the cycle of SDS22-PP1-I3 formation and disassembly recycles PP1 between holophosphatase complexes and raises the possibility that this cycle may accelerate the subunit exchange on PP1 in cells. Rebinding of SDS22 and I3, however, is relatively slow in vitro suggesting additional cellular mechanisms exist to speed up SDS22-PP1-I3 formation and maintain the steady state between formation and disassembly.

As a crucial part of the model above, in this study, we also demonstrate directly that the SDS22-PP1-I3 population indeed can serve as a pool for the formation of active PP1 holoenzymes. Using reconstitution from purified proteins, we show that SDS22-PP1-I3 disassembly by p97 releases active PP1 that can bind a substrate-specifying subunit to dephosphorylate a specific protein substrate. This holophosphatase is labile, though, because once p97 is inhibited, SDS22 and I3 snap back onto PP1 thus displacing the activating subunit. Consistent with that notion, in cells, inhibition of p97 leads to sequestration of free SDS22 and I3 and formation of SDS22-PP1-I3 concomitant with a drop in cellular PP1 activity. This is in accordance with an early observation that the p37 homolog Shp1 is essential for PP1/Glc7 activity in yeast (25, 26) and supports the notion that the SDS22-PP1-I3 complex is thermodynamically favored.

Structurally, binding of SDS22 and I3 to PP1 coincides with loosening of the catalytic metal ions in PP1 (12, 13). This could mean that SDS22 and I3 trap and stabilize metal-deficient PP1 that lost a metal ion (27) or help exchange an incorrectly loaded metal ion which is known to affect specificity (28). A mutually nonexclusive possibility is that destabilizing ion binding is part of the inhibitory effect to ensure that PP1 stays inactive in the SDS22-PP1-I3 pool. If so, metal ion rebinding to PP1 occurs quickly and spontaneously as we show that PP1 released in vitro is active.

Based on these results, we propose that a fine-tuned balance between SDS22 and I3 binding and p97-mediated disassembly maintains a dynamic PP1 holoenzyme system with dynamic exchange of subunits that keeps PP1 activity under control and, at the same time, ensures responsiveness to varying cellular demands in the context of a cellular excess of regulatory subunits over PP1 catalytic subunits.

## Material and Methods

### Plasmids.

pFL insect cell expression plasmids for human PP1γ and SDS22, and I3 were described before (5). pFL-PP1-Clover was created from the plasmids pCAGGS-Raichu-Rho-CR (29) using Gibson cloning. Sortase sites were inserted for pFL-I3-LPETGGG, GGG-SDS22, and NIPP1-LPETGGG (original NIPP1 sequence from ref. 30). pET16b-PP1 for bacterial expression was created by Gibson cloning from previously described constructs (5). PP1γ^H66K^ was created via site-directed mutagenesis and cloned into pST44. pMAL-hGADD34^513-636^, pGEX-4 T-1-PERK KD, pSUMO3-1-heIF2α^2-187^ were described before (21, 23, 31). pEGFP-N1-hGADD34 was a kind gift from Stefan J. Marciniak (University of Cambridge, Cambridge, UK) (32). pET30b-7 M-SortaseA and pET28a-SENP2 were kind gifts from H. Ploegh (Harvard Medical School, Cambridge, MA, US) (Addgene #51141) and Guy Salvesen (Sanford-Burnham Institute, La Jolla, CA, US) (Addgene plasmid # 16357). Full-length GADD34-GFP was inserted into pDONR221 and pcDNA5-FRT for creation of a stable cell line.

### Protein Expression and Purification.

The following proteins were expressed in High Five insect cells (Thermo). All protein complexes were coexpressed. The PP1-I3 (PI) and SDS22-PP1-I3 (SPI) complexes and variants thereof were generated as before (5). In brief, untagged SDS22 and PP1γ (or variants) were coexpressed with His-I3 (or variants). This was achieved using a virus harboring a dual-expression cassette encoding SDS22 and PP1γ, together with a second virus encoding His-I3. The PI and SPI complexes were purified on a HisTrap FF (Cytiva) column in p97 buffer (50 mM 2-[4-(2-hydroxyethyl)piperazin-1-yl]ethanesulfonic acid (HEPES) pH 8, 150 mM KCl, 5 mM MgCl_2_, 5% glycerol) with 25 mM imidazole and eluted directly onto a HiTrap Q HP anion exchange column 5 mL (Cytiva) with p97 buffer plus 300 mM imidazole. After elution in p97 buffer with 1 M KCl, proteins were run on a Superdex 16/600 pg 200 (Cytiva) in p97 buffer with 1 mM dithiothreitol (DTT). His-PP1-SDS22 (SP) was generated as above omitting the anion exchange chromatography step. His-p97 was eluted from a HisTrap FF directly onto a HiTrap Q HP 5 column and buffer was exchanged by repeated concentration and dilution in p97 buffer with 1 mM DTT. G-SDS22 was generated in Hi5 cells as described above. His-tagged Gly-SDS22 (with N-terminal glycine) was purified from cleared High Five cell lysates in lysis buffer (50 mM HEPES pH 8.0, 0.5 M KCl, 5 mM imidazole, 0.1% Triton X-100). Lysate was loaded on a HisTrap FF (Cytiva) column in lysis buffer containing 5 mM imidazole and no Triton. Column was washed until a base line was reached followed by a wash with 8% elution buffer (50 mM HEPES pH 8.0, 0.5 M KCl, 250 mM imidazole). Column was incubated overnight with wash buffer containing 1,000 units His-tagged Tobacco Etch Virus (TEV)-protease (Sigma-Aldrich), to remove the His-tag and reveal an N-terminal glycine for sortase-labeling. The protein was eluted with wash buffer. His-PP1γ:SDS22 (SP) and His-PP1γ:NIPP1 (NP) complexes were expressed and purified with respective labels as described before for the SPI complexes. Same buffers and procedures were used, but the anion exchange chromatography was omitted from the protocol. Of note, PP1 was tagged with a 6-histidine tag in both complexes.

The following proteins were expressed in *Escherichia coli* BL21 (DE3) (Agilent). His-NIPP1-LPETGGG, His-SENP2, and His-Sortase A were purified using HisTrap FF 5 mL (Cytiva) column as described above, followed by a Superdex 16/600 pg 75 (Cytiva) in p97 buffer with 1 mM DTT. GST-PERK catalytic domain was purified on a GSTrap HP column (Cytiva) in GST buffer (50 mM HEPES pH 7.5, 150 mM KCl, 1 mM DTT) and eluted with 20 mM glutathione. eIF2α^2-187^ was expressed as an N-terminally small ubiquitin-like modifier (SUMO)- and His-tagged protein and purified on a HisTrap FF column (Cytiva) in wash buffer [50 mM HEPES pH 7.5, 500 mM KCl, 10 mM imidazole, 0.5 mM tris(2-carboxyethyl)phosphine (TCEP)]. Elution was with 300 nM imidazole. SUMO-tag cleavage with 0.01 mg/mL His-SENP2 protease was conducted overnight at 4 °C. eIF2α^2-187^ was collected by elution with wash buffer. MBP-His-GADD34^513-636^ and MBP-His were purified on a HisTrap FF (Cytiva) column in lysis buffer (50 mM Tris pH 7.4, 500 mM NaCl, 20 mM imidazole, 0.5 mM TCEP) followed by MBP-purification using an MBPTrap HP 5 mL (Cytiva) and gel filtration on a Superdex 26/600 pg 200 column (Cytiva). p37 was generated from GST-p37 with GST tag removal by PreScission protease (GE) as described previously (5).

Actin from rabbit muscle (Sigma-Aldrich) was diluted to 1 mg/mL in G-buffer (5 mM Tris pH 8.0, 0.2 mM ATP, 0.5 mM DTT, and 0.2 mM CaCl_2_). PP1 and PP1^H66K^ were generated in *E. coli* ArcticExpress DE3 (Agilent) as described before (33).

### Protein Labeling.

TAMRA [5(6)-carboxy-tetramethylrhodamin] labeling of His-NIPP1-LPETGGG, GGG-SDS22-PP1^Clover^-His-I3, SDS22-PP1^Clover^-His-I3-LPETGGG, PP1^Clover^-His-I3-LPETGGG, and GGG-His-SDS22-PP1^Clover^ was performed by using a Sortase A heptamutant (Ca^2+^ independent). His-NIPP1-LPETGGG, SDS22-PP1^Clover^-His-I3-LPETGGG, PP1^Clover^-His-I3-LPETGGG (50 μM) were incubated with TAMRA-conjugated Strep-tag peptide GGGWSHPQFEKK-TAMRA (200 μM) and Sortase A (2.5 μM) in p97 buffer with 1 mM DTT at 30 °C for 2 h. For GGG-SDS22-PP1^Clover^-His-I3, and GGG-His-SDS22-PP1^Clover^ the TAMRA-conjugated Strep-tag peptide (TAMRA)-AWSHPQFEKLPETGGG was used instead. Peptides were synthesized and labeled by Caslo, Denmark. Labeled proteins were purified via the strep-tag in the TAMRA peptide using a StrepTrap HP (Cytiva) column followed by gel filtration on a Superdex 16/600 pg 200 (Cytiva) column or Superdex 16/600 pg 75 (Cytiva) column for NIPP1, in p97 buffer with 1 mM DTT.

### In Vitro eIF2α Phosphorylation with PERK.

50 µM eIF2α^2-187^ and 100 nM GST-PERK catalytic domain were incubated at 37 °C for 2 h in a total volume of 4 mL containing 100 mM KCl, 25 mM HEPES pH 7.5, 5 mM MgCl_2,_ 1 mM TCEP, and 2.5 mM ATP according to ref. 23. GST-PERK was removed on a GSTrap HP column (Cytiva) as described above.

### FRET-Based Assays.

All time course measurement samples (72 μL) were diluted in p97 buffer + 2% bovine serum albumin (BSA)+ 1 mM DTT, transferred to fluorescence cuvettes (Hellma) and heated to 30 °C. For kinetics, a baseline was recorded for 3 to 5 min before the reaction was started by the addition of ATP (2 mM). Samples were excited at a wavelength of 475 ± 10 nm. Donor emission was recorded at 518 nm and acceptor emission at 580 ± 2.5 nm. For data analysis, we normalized each trace’s fluorescence intensity before addition of ATP (8 μL) to remove the dilution effect. The ratio of both emission intensities was calculated by dividing the acceptor intensity by the donor intensity. Curves were normalized between the FRET signal before the addition of ATP and the lowest (dissociation/disassembly) or the highest plateau (association) at the end of the reaction in the dataset. p97 inhibition was induced by adding 10 µM CB-5038 (Selleckchem). Cuvettes were mixed by gently pipetting up and down. Data points collected while adding the inhibitor or dimethyl sulfoxide (DMSO) were removed. Unless otherwise stated, reactions contained: ATP (2 mM), p97 (160 nM), p37 (480 nM), SDS22-PP1γ^Clover^I3 versions (160 nM), and NIPP1 or GADD34 versions (240 nM) based on previously determined optimal assay conditions (18).

In disassembly reactions with PP1^Clover^-I3^TAMRA^ (PI) (160 nM) free SDS22 was added at 160 nM.

### Fluorometric OMFP Dephosphorylation Assay.

OMFP was from Novachemistry, UK, and stored as a 100 mM stock in DMSO +100 mM H_2_SO_4_. OMFP dephosphorylation was monitored using a Varian Cary Eclipse spectrofluorometer (Thermo). Excitation was at 485 nm (5 nm slit width). Emission was recorded at 525 nm (5 nm slit width). Reactions (120 µL) contained OMFP (100 µM), SDS22-PP1-I3 (160 nM), p97 (160 nM), p37 (480 nM), and ATP (2 mM) in p97 buffer with 1 mM DTT unless otherwise stated. First, a baseline was recorded for 3 to 4 min of only OMFP, while all other proteins were preincubated in parallel. Then, the mix containing all other components was supplemented with ATP, added to the OMFP and the reactions were recorded over a time course of 60 min. Incubation of inactive PP1^H66K^ with SDS22-PP1-I3 (SPI) with p97 but without p37 (Δp37) did not lead to activation of PP1, confirming no subunit exchange of SDS22 and I3 with the inactive sponge. OMFP background hydrolysis was recorded and deducted from all other traces. apo-PP1 (160 nM) was used as a positive control to ensure OMFP reactivity with PP1. Δp37 and ΔSPI controls contained 480 nM PP1^H66K^.

### eIF2α Dephosphorylation Assay and PhosTag gel Analysis.

Actin was preincubated with a 10× molar excess of latrunculin B (Sigma-Aldrich) for 1 h on ice to ensure actin depolymerization. Reactions (20 µL) contained p37 (480 nM), SPI (160 nM), MBP-GADD34^513-636^ (400 nM), G-actin (400 nM), eIF2α^P^ (2,000 nM), ATP (2 mM), and p97 (1, 2, 5, 10, 20 nM) in assay buffer (50 mM HEPES pH 8.0, 200 mM KCl, 0.02% Triton, 2 mM MnCl_2_, and 1 mM DTT) adapted from ref. 22. Reactions were started by adding ATP and incubated at 30 °C for 10 min. Reactions were stopped by adding 5 µL 6× SDS-loading buffer. Samples were analyzed using 12% polyacrylamide gels containing 5 mM PhosTag AAL solution (Fujifilm) and 10 mM MnCl_2_. Band intensities were quantified using ImageJ software and normalized to ΔGADD34 sample, and shown as percentages of total eIF2α-P and eIF2α.

### Cell Culture.

A stably inducible HeLa GADD34-GFP cell line was generated with the FlpIn T-Rex system (Thermo) according to the manufacturer’s instructions and single clones were selected. The HeLa GADD34-GFP cell line was cultured in Dulbecco’s modified Eagle’s medium supplemented with 10% fetal bovine serum supplemented with penicillin/streptomycin (100 µg/mL), hygromycin (250 µg/mL), and blasticidine (15 µg/mL) at 37 °C and 5% CO2. Sf9 and Hi5 insect cells were cultivated in suspension in Spodopan (PAN Biotech) at 27 °C with continuous shaking at 120 rpm.

### Coimmunoprecipitations.

For GADD34 IPs, GADD34 expression in the HeLa GADD34-GFP cell line was induced with doxycycline (1 µg/mL) for 2 h. Subsequently, CB-5083 (5 µM) was added for 3 h. For IP of endogenous PP1, HeLa cells were treated with CB-5083 for 70 min.

Cells were then lysed in lysis buffer [50 mM KCl, 50 mM Tris at pH 7.4, 5 mM MgCl_2_, 5% glycerol, 1% Triton X-100, 2 mM β-mercaptoethanol, complete EDTA-free protease inhibitor (Roche), and PhosSTOP (Roche)]. Pull-downs were performed with GFP nanobody slurry (GADD34 IPs) or PP1γ antibody with protein G beads (PP1 IPs) in lysis buffer for ~2 h at 4 °C. Beads were eluted by boiling in SDS-loading buffer.

### Disassembly Reactions Followed by Coimmunoprecipitations.

p97 (160 nM), p37 (480 nM), SDS22-PP1^Clover^-I3 (160 nM), and NIPP1^TAMRA^ (480 nM) were incubated in p97 buffer with DTT (1 mM) and BSA (2%). Disassembly was initiated by addition of ATP (2 mM) and incubation at 30 °C. 10 µM CB-5038 (Selleckchem), 0.5 U apyrase, or DMSO as a control was added after 10 or 30 min, Reactions were stopped by tenfold dilution in ice-cold p97 buffer with Triton X-100 (1%) and BSA (2%) and incubated with PP1γ antibody (or goat IgG as control) and GammaBind G Sepharose (Cytiva) for 1 h at 4 °C. Beads were eluted by boiling in SDS-loading buffer.

### Antibodies.

Rabbit polyclonal anti-NIPP1 (Sigma Cat. no. HPA027452, 1:500), goat polyclonal anti-SDS22 (Santa Cruz E-20, Cat. no. sc-162164), mouse monoclonal anti-SDS22 (B-6) (Santa Cruz, Cat. no. sc-398864, 1:1,000), rabbit polyclonal anti-I3 [(34), 1:1,000], mouse monoclonal anti-GFP (Roche, Cat. no. 11814460001, 1:500), mouse monoclonal anti-PP1α (E-9) (Santa Cruz C-19, Cat. no. sc-7482, 1:500), mouse monoclonal anti-PP1γ (Santa Cruz E-4, Cat. no. sc-515943, 1:1,000), goat polyclonal anti-PP1γ (Santa Cruz C-19, Cat. no. sc-6108 1:500), rabbit polyclonal anti-KNL1 [(35), 1:750], rabbit polyclonal anti-URI1 (New England Biolabs, Cat. no. 5844, 1:1,000), mouse monoclonal anti-puromycin (Sigma Cat. no. MABE343, 1:20,000), rabbit polyclonal anti-PPP1R15B (CReP) (Proteintech Cat. no. 14634-1-AP, 1:2,000), mouse monoclonal anti-α-tubulin (Sigma Cat. no. T-5168, 1:10,000).

### Analytical Gel Filtrations.

HEK293 cells were treated with CHX (90 µg/mL) and CB-5083 (10 µM) for 70 min. Cells were lysed in lysis buffer [50 mM KCl, 50 mM Tris at pH 7.4, 5 mM MgCl_2_, 5% glycerol, 1% Triton X-100, 2 mM β-mercaptoethanol, complete EDTA-free protease inhibitor (Roche), and PhosSTOP (Roche)]. Cleared lysates were separated by size exclusion chromatography (SEC) on a Superdex 200 10/300 column (Cytiva) in SEC buffer (50 mM HEPES pH 7.4, 150 mM NaCl) at a flow rate of 0.4 ml/min with 500 µL fraction size. Fractions were analyzed by sodium dodecyl sulfate-polyacrylamide gel electrophoresis (SDS-PAGE) and western blotting with indicated antibodies. Band intensities were quantified and divided by the sum of intensities to obtain the relative amount in each lane.

### Puromycin Incorporation Assay.

HEK293 cells were seeded in a 6-well plate and treated with DMSO as control or 90 µg/mL CHX for the indicated time points. Cells were pulsed additionally with 10 µg/mL puromycin for 10 min. Cells were lysed in lysis buffer and samples were normalized for protein concentration prior to analysis by western blotting.

### PP1 Activity Assay.

HeLa cells constitutively expressing GFP-PP1 (36) were treated with CB-5083 (10 µM) and CHX (90 µg/mL) for 120 min. Cells were briefly sonicated in lysis buffer [50 mM KCl, 50 mM Tris at pH 7.4, 5 mM MgCl_2_, 5% glycerol, 1% Triton X-100, 2 mM β-mercaptoethanol, complete EDTA-free protease inhibitor (Roche), and PhosSTOP (Roche)] using a Bioruptor (Diagenode; 30 s on, 30 s off, five cycles, high intensity) and GFP-PP1 was isolated from cleared lysates with anti-GFP nanobodies coupled to agarose beads. Beads were incubated with 133 nM OMFP in p97 buffer at 30 °C and OMF fluorescence determined in a Varian Cary Eclipse spectrofluorometer. Subsequently, GFP-PP1 was eluted by boiling in Laemmli sample buffer, samples were analyzed by SDS-PAGE and western blotting for quantification of GFP band. The OMFP dephosphorylation activity was normalized to the amount of GFP-PP1.

## Supplementary Material

Appendix 01 (PDF)

## Data Availability

All study data including original scans and raw data have been deposited at Mendeley Data and are publicly available as of the date of publication (https://dx.doi.org/10.17632/vw42rzt2c2.1) (37). All other data are included in the article and/or *SI Appendix*.
