# Supplementary material for: Alternating binding and p97-mediated dissociation of SDS22 and I3 recycles active PP1 between holophosphatases
Source: Proc Natl Acad Sci U S A. 2024 Aug 29;121(36):e2408787121. doi: 10.1073/pnas.2408787121 (PMC11388335; doi:10.1073/pnas.2408787121)
Supplement: Supplementary file 1 — Appendix 01 (PDF) [file pnas.2408787121.sapp.pdf]

PNAS

Supporting Information for
Alternating binding and p97-mediated dissociation of SDS22 and I3 recycles active PP1 between holophosphatases

Anja F. Kueck, Johannes van den Boom, Sandra Koska, David Ron, Hemmo Meyer

Hemmo Meyer
Email: hemmo.meyer@uni-due.de

This PDF file includes:

Figures S1 to S5

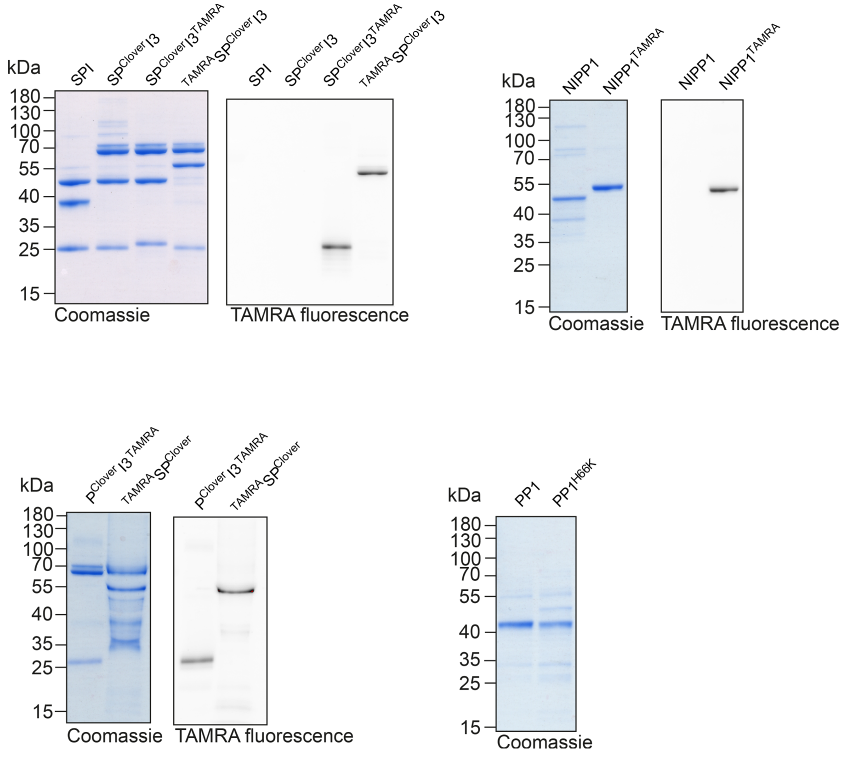

Fig. S1. Protein preparations for in vitro assays.

SDS-gels of indicated proteins or complexes. Detection by Coomassie and TAMRA fluorescence of the same gels.

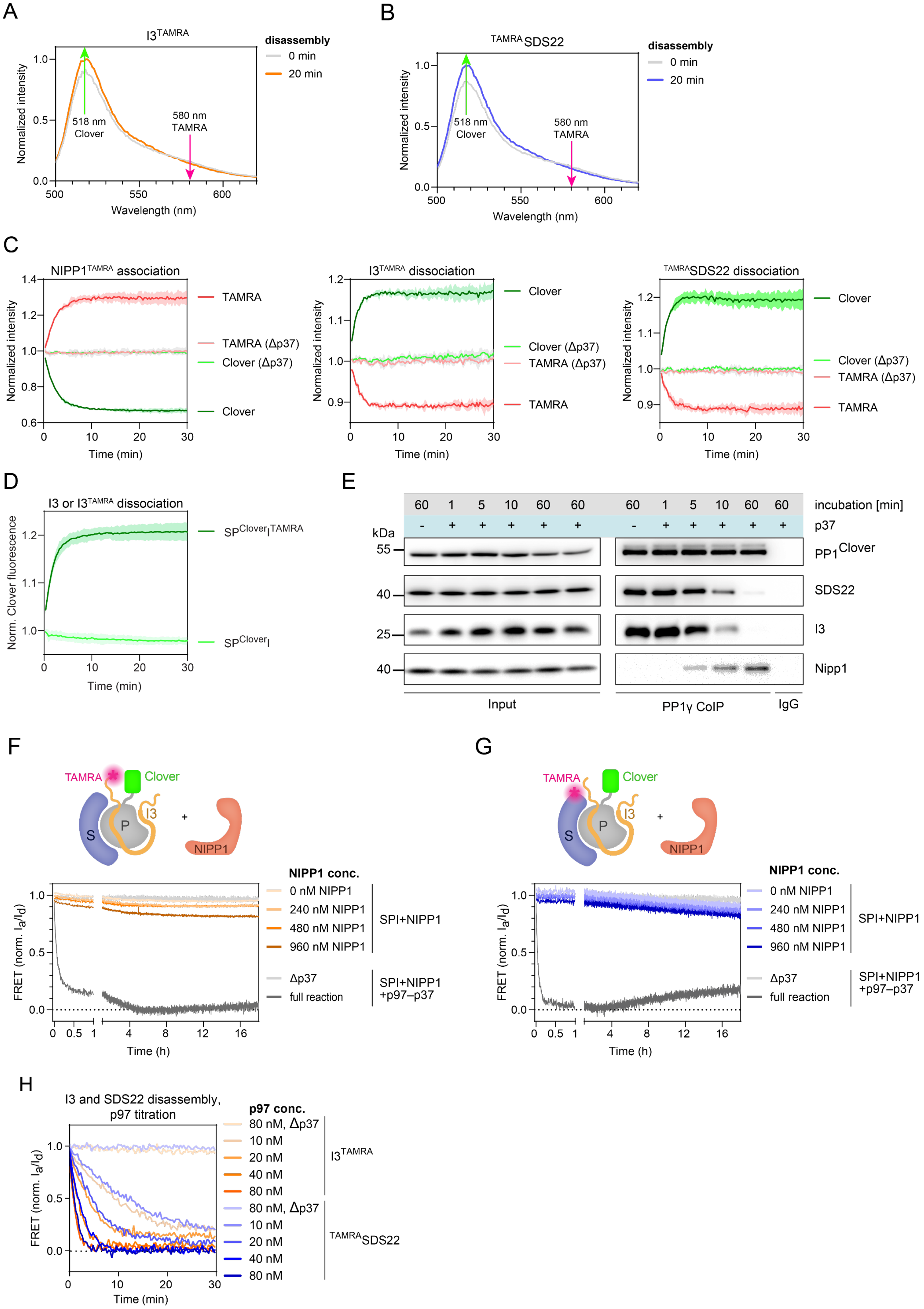
A
I3TAMRA
disassembly
0 min
20 min
Normalized intensity
518 nm Clover
580 nm TAMRA
Wavelength (nm)
B
TAMRASDS22
disassembly
0 min
20 min
Normalized intensity
518 nm Clover
580 nm TAMRA
Wavelength (nm)
C
NIPP1TAMRA association
TAMRA
TAMRA (Δp37)
Clover (Δp37)
Clover
Normalized intensity
Time (min)
I3TAMRA dissociation
Clover
Clover (Δp37)
TAMRA (Δp37)
TAMRA
TAMRASDS22 dissociation
Clover
Clover (Δp37)
TAMRA (Δp37)
TAMRA
D
I3 or I3TAMRA dissociation
SPCloverITAMRA
SPCloverI
Norm. Clover fluorescence
Time (min)
E
incubation [min]
p37
kDa
PP1Clover
SDS22
I3
Nipp1
Input
PP1γ CoIP
IgG
F
TAMRA
Clover
S
P
I3
NIPP1
NIPP1 conc.
0 nM NIPP1
240 nM NIPP1
480 nM NIPP1
960 nM NIPP1
SPI+NIPP1
Δp37
full reaction
SPI+NIPP1 +p97–p37
FRET (norm. Ia/Id)
Time (h)
G
TAMRA
Clover
S
P
I3
NIPP1
NIPP1 conc.
0 nM NIPP1
240 nM NIPP1
480 nM NIPP1
960 nM NIPP1
SPI+NIPP1
Δp37
full reaction
SPI+NIPP1 +p97–p37
FRET (norm. Ia/Id)
Time (h)
H
I3 and SDS22 disassembly, p97 titration
p97 conc.
80 nM, Δp37
10 nM
20 nM
40 nM
80 nM
I3TAMRA
80 nM, Δp37
10 nM
20 nM
40 nM
80 nM
TAMRASDS22
FRET (norm. Ia/Id)
Time (min)

Fig. S2. Characterization of FRET assay for subunit exchange (related to Fig. 1).
(A) Fluorescence emission spectra before and after a subunit exchange reaction with the ^{TAMRA}SDS22-PP1^{Clover}-I3 complex, in which PP1 is fused to Clover and SDS22 coupled to TAMRA, and unlabeled NIPP1 catalyzed by p97-p37. FRET donor (Clover) and acceptor (TAMRA) emission intensity shifts are indicated. Representative spectrum of 3 experiments with similar outcome.
(B) As in (A) but with SDS22-PP1^{Clover}-I3^{TAMRA} in which TAMRA is coupled to I3.
(C) Fluorescence intensities of Clover (FRET donor) and TAMRA (FRET acceptor) signal of the curves presented in Figure 1 A-C. Intensities were normalized to the signal before addition of ATP. N=3 ± SD.
(D) Change in Clover signal depends on FRET with TAMRA acceptor. SDS22-PP1^{Clover}-I3 was incubated with p97 (160 nM), p37 adapter (480 nM), NIPP1 (240 nM) and ATP (2 mM) in the absence of TAMRA label. Reactions with SDS22-PP1^{Clover}-I3^{TAMRA} served as control. N=4 ± SD.
(E) A subunit exchange reaction as in Fig. 1 A was carried out with NIPP1. PP1 was immuno-isolated at indicated times and associated SDS22 and I3 detected by Western blotting. Representative blot of 3 replicates with similar outcome.
(F and G) Long-term FRET measurement as in Fig. 1 (A-C) of SDS22-PP1^{Clover}-I3^{TAMRA} (F) and ^{TAMRA}SDS22-PP1^{Clover}-I3 (G) and, respectively, in the presence of increasing concentrations of unlabeled NIPP1. Note that the SDS22-PP1-I3 complex (160 nM) does not significantly disassemble over the course of 18 h even at 6-fold excess of NIPP1 if p97-p37 is not proficient. Representative graphs of 2 experiments with similar outcome.
(H) SDS22 and I3 dissociate simultaneously over a range of complex disassembly rates at different p97 concentrations. FRET assays as in Fig. 1 (A-C) with indicated protein combinations. Representative graphs of 2 experiments with similar outcome.

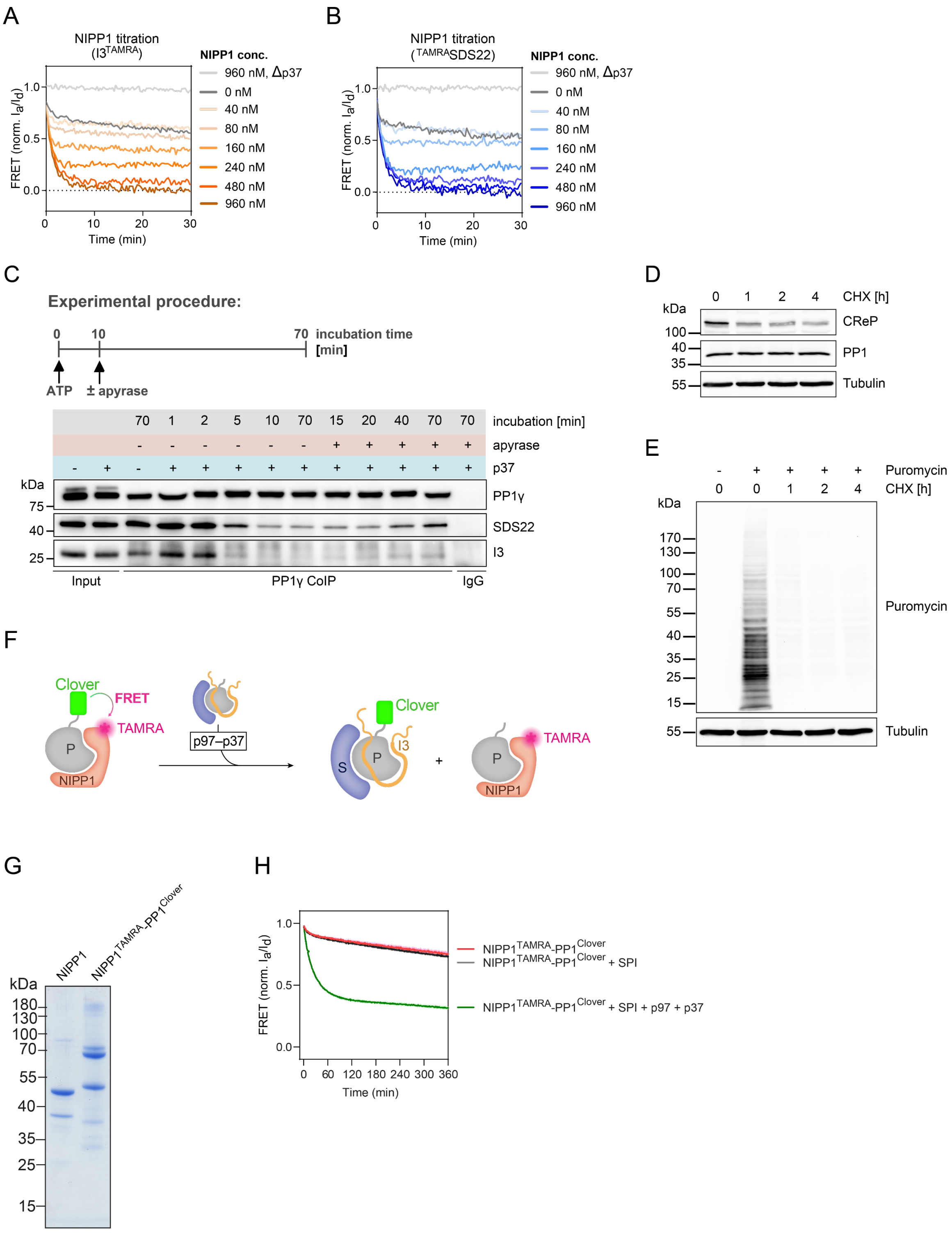
A
NIPP1 titration (I3TAMRA)
FRET (norm. Ia/Id)
Time (min)
NIPP1 conc.
960 nM, Δp37
0 nM
40 nM
80 nM
160 nM
240 nM
480 nM
960 nM
B
NIPP1 titration (TAMRASDS22)
FRET (norm. Ia/Id)
Time (min)
NIPP1 conc.
960 nM, Δp37
0 nM
40 nM
80 nM
160 nM
240 nM
480 nM
960 nM
C
Experimental procedure:
incubation time [min]
ATP
± apyrase
incubation [min]
apyrase
p37
kDa
PP1γ
SDS22
I3
Input
PP1γ CoIP
IgG
D
kDa
CHX [h]
CReP
PP1
Tubulin
E
Puromycin
CHX [h]
kDa
Puromycin
Tubulin
F
Clover
FRET
TAMRA
P
NIPP1
p97–p37
S
I3
G
NIPP1
NIPP1TAMRA-PP1Clover
kDa
H
FRET (norm. Ia/Id)
Time (min)
NIPP1TAMRA-PP1Clover
NIPP1TAMRA-PP1Clover + SPI
NIPP1TAMRA-PP1Clover + SPI + p97 + p37

Fig. S3. Net SDS22-PP1-I3 disassembly by p97 depends on the concentration of the alternative subunit NIPP1.

(A and B) The FRET subunit exchange reaction was carried out with SDS22-PP1^{Clover}-I3^{TAMRA} or ^{TAMRA}SDS22-PP1^{Clover}-I3, respectively, with indicated increasing concentrations of unlabeled NIPP1. Representative graphs of 2 experiments with similar outcome.

(C) A subunit exchange reaction similar to Fig. 2 (B and C) was carried out. After 10 min, p97 was inhibited by ATP depletion (apyrase). PP1 was immuno-isolated at indicated times and associated SDS22 and I3 detected by Western blotting. Representative blot of 4 replicates with similar outcome.

(D) Confirmation of effect of cycloheximide (CHX). HEK293 cells were treated with 90 µg/ml cycloheximide for indicated times. Whole cell lysates were probed by Western blot with indicated antibodies. The unstable protein CReP served as positive control.

(E) Additional confirmation of cycloheximide activity. HEK293 cells were treated with 90 µg/ml cycloheximide and pulsed with 10 µg/ml puromycin or vehicle control for the last 10 min as indicated. Puromycylated nascent polypeptides were visualized by Western blot.

(F) Model for p97-mediated subunit exchange of the NIPP1-PP1 complex.

(G) SDS gel analysis of NIPP1^{TAMRA}-PP1^{Clover} complex purified from insect cells used in the NIPP1-PP1 disassembly assay.

(H) Disassembly of the NIPP1^{TAMRA}-PP1^{Clover} complex by SDS22-PP1-I3 measured by FRET subunit exchange. NIPP1^{TAMRA}-PP1^{Clover} (80 nM) was incubated without or with p97 (160 nM), p37 (480 nM) and unlabeled SDS22-PP1-I3 (80 nM) or unlabeled NIPP1 (80 nM), as indicated. Reactions were started by addition of ATP (2 mM). Zero FRET value of fully disassembled NIPP1^{TAMRA}-PP1^{Clover} was determined by incubation with 100-fold excess of unlabeled NIPP1 until the signal plateaued. N=4 (N=3 for NIPP1^{TAMRA}-PP1^{Clover} alone) ± SD.

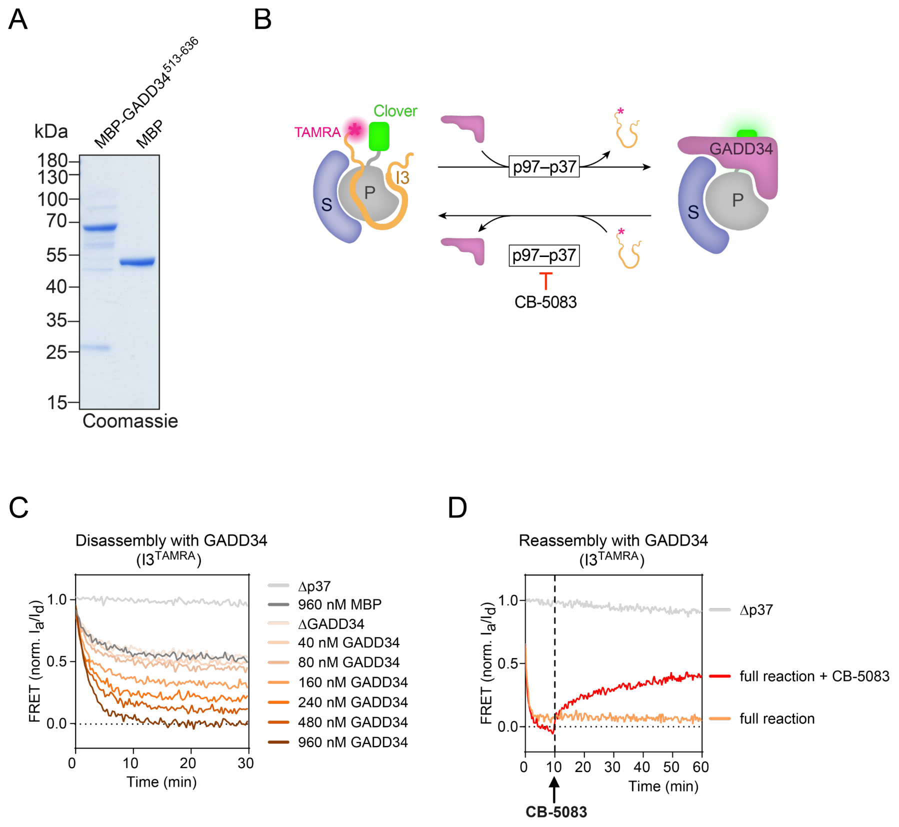

Fig. S4. GADD34 binds PP1 released from SDS22-PP1-I3 by p97.

(A) SDS gel analysis of MBP-GADD34 and MBP used in the subunit exchange assay.

(B) Model for p97-mediated subunit exchange with the substrate specifier GADD34.

(C) FRET subunit exchange reactions with SDS22-PP1^{Clover}-I3^{TAMRA}, as in Fig. 1B, in the presence of indicated increasing concentrations of unlabeled GADD34. Note that GADD34 increased net disassembly of SDS22-PP1-I3, demonstrating binding of GADD34 to PP1.

(D) Disassembly reactions with SDS22-PP1^{Clover}-I3^{TAMRA} in the presence of GADD34 were stopped after 10 min by CB-5083. Note that I3 re-associates with PP1 after p97 inhibition. Representative graphs of 3 experiments with similar outcome.

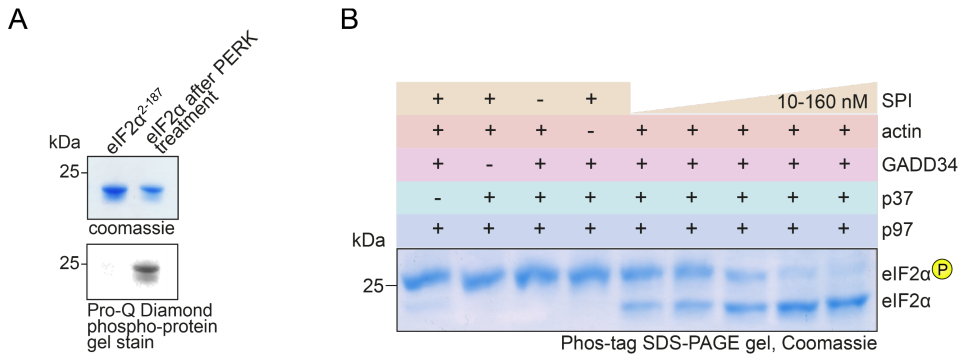

Fig. S5. Experiments related to eIF2α dephosphorylation.

(A) Phosphorylation of eIF2α. eIF2α was expressed in bacteria, phosphorylated by PERK and reisolated. Confirmation of phosphorylation by a Pro-Q Diamond phospho-protein gel stain.

(B) Coupled PP1 subunit exchange and eIF2α dephosphorylation reactions as in Fig. 4B but with varying concentrations of SDS22-PP1-I3 (SPI) as indicated. Representative gel of 2 replicates with similar outcome.
